# Reprogramming of lipid metabolism in the tumor microenvironment: a strategy for tumor immunotherapy

**DOI:** 10.1186/s12944-024-02024-0

**Published:** 2024-02-01

**Authors:** Yuting Wu, Xi Pu, Xu Wang, Min Xu

**Affiliations:** 1 Department of Gastroenterology, Jiangsu University Cancer Institute, Affiliated Hospital of Jiangsu University, 438 Jiefang Road, Jingkou, Zhenjiang, Jiangsu 212001 P. R. China; 2 Department of Radiation Oncology, Institute of Oncology, Affiliated Hospital of Jiangsu University, Zhenjiang, 212001 Jiangsu China; 3 Digestive Disease Research Institute of Jiangsu University, Zhenjiang, 212001 Jiangsu China; 4 Department of Radiation Oncology, Jiangsu University Cancer Institute, Affiliated Hospital of Jiangsu University, 438 Jiefang Road, Jingkou, Zhenjiang, Jiangsu 212001 P. R. China

**Keywords:** Lipid metabolism, Tumor microenvironment, Immunotherapy, Programmed cell death protein 1, Targeted therapy

## Abstract

Lipid metabolism in cancer cells has garnered increasing attention in recent decades. Cancer cells thrive in hypoxic conditions, nutrient deficiency, and oxidative stress and cannot be separated from alterations in lipid metabolism. Therefore, cancer cells exhibit increased lipid metabolism, lipid uptake, lipogenesis and storage to adapt to a progressively challenging environment, which contribute to their rapid growth. Lipids aid cancer cell activation. Cancer cells absorb lipids with the help of transporter and translocase proteins to obtain energy. Abnormal levels of a series of lipid synthases contribute to the over-accumulation of lipids in the tumor microenvironment (TME). Lipid reprogramming plays an essential role in the TME. Lipids are closely linked to several immune cells and their phenotypic transformation. The reprogramming of tumor lipid metabolism further promotes immunosuppression, which leads to immune escape. This event significantly affects the progression, treatment, recurrence, and metastasis of cancer. Therefore, the present review describes alterations in the lipid metabolism of immune cells in the TME and examines the connection between lipid metabolism and immunotherapy.

## Introduction

Lipids are composed of fatty acids, triglycerides, cholesterol, cholesteryl esters, phospholipids and sphingolipids and are widely distributed in cells. Lipids form cell membranes and serve as an energy source for cells. Lipids also function as secondary messengers to transmit signals and mediate cell growth, proliferation, apoptosis and death [1]. Due to their rapid growth, tumors develop areas of nutrient deprivation and hypoxia. To adapt to nutrient-deficient and hypoxic environments, tumor cells undergo lipid reprogramming to accelerate malignant behavior and increase glucose uptake and glycolysis. Increased fatty acid oxidation, lipid production, lipid absorption, and lipid storage are found in cancer cells [1]. Lipids contribute to tumor cell growth, invasion, metastasis, radiation and chemotherapy resistance. Fats encourage tumor cells to preserve their stemness by activating the Wnt and Notch signaling pathways [2, 3].

The unusually complex TME is composed of immune cells, fibroblasts and tumor-associated endothelial cells. Immune cells include tumor-associated macrophages (TAMs), tumor-infiltrating T cells (TILs), dendritic cells (DCs), myeloid-derived suppressor cells (MDSCs), and natural killer (NK) cells [4]. Cancer cells survive in an environment that changes as a result of altered lipid metabolism. Different immune cells have different metabolic preferences. For example, M1-TAMs prefer glycolysis, whereas M2-TAMs prefer lipid peroxidation [5]. The effect of lipids on immune cells is also complex. Excessive fatty acid and cholesterol accumulation leads to a functional decline in CD8 + T cells. Cytokine levels are decreased, and the anti-tumor effect of T cells is weakened [6]. However, linoleic acid (LA) and short-chain fatty acids (SCFAs) exert opposite effects. These factors enhance T-cell activity and encourage CD8 + T cells to convert into memory T cells [7].

Immunotherapy, radiation therapy, and chemotherapy are currently used to treat cancers. Numerous cell signaling pathways lead to resistance to radiation and chemotherapy, including cell survival, proliferation, anti-apoptosis, invasion and metastasis pathways. Lipid metabolites are responsible for malignant progression and radiotherapy resistance [8]. For example, arachidonic acid produces enzymes, such as cytosolic phospholipase A2, cyclooxygenase and lipoxygenase, which cause tumor radiotherapy resistance. Many efforts have been made to identify the machinery associated with lipid metabolites and the radiation signals leading to radiation resistance [9].

A number of cytokines and inflammatory mediators are crucial for the development of tumors, autoimmune disorders, infections, and other illnesses. These mediators are involved in information transmission, immune cell activation and regulation, and T and B cell activation [10]. For example, interleukin (IL)-11 aggravates autoimmune encephalomyelitis. IL-11 increases the number of inflammatory foci in the spinal cord and the number of IL-17 + CD4 + cells. It may be used as a therapeutic target for multiple sclerosis [11]. IL-11 induces the secretion of NLRP3 inflammasome-related genes and IL-1β in monocytes. It also regulates monocyte migration to the central nervous system in relapsing–remitting multiple sclerosis [12]. The innate and adaptive immune systems play a role in host defense against cancer via various mechanisms, which has driven an unprecedented development of modern cancer immunotherapy. Immunotherapy is the most promising anti-tumor medicine on the market. Compared to patients receiving conventional medication, individuals who respond to immunotherapy have a greater probability of long-term survival with fewer adverse effects [13]. Tumor-targeted monoclonal antibodies, cytokine and immune checkpoint inhibitor (ICI) therapies, chimeric antigen receptor-T (CAR-T) cells, and cancer vaccines are examples of immunotherapies. For example, antibodies against programmed cell death protein 1 (PD-1) reverse the antitumor effects of T cells in certain settings. However, only a few patients benefit from this approach. Tumor cells that undergo metabolic reprogramming in the TME are more likely to elude immune surveillance, which results in a state of immunosuppression and decreased sensitivity to PD-1 antibodies [14]. Tumor lipid reprogramming encourages immune cell malfunction to defeat immune surveillance systems. Small molecule drugs that target lipid metabolism in combination with immunotherapy has achieved better therapeutic effects. This combination therapy improves sensitivity to radiotherapy and chemotherapy [15]. The novelty of our review is the comprehensive description of lipid metabolism reprogramming in the tumor microenvironment. We also mention the current drugs that target lipid metabolism, which provide novel approaches for tumor treatment.

### Lipid metabolism processes

Noticeable changes are often observed in lipid metabolism in cells that have transitioned to a malignant phenotype. Fatty acid (FA) absorption, FA oxidation (FAO) and de novo lipogenesis can all be increased by tumor cells in order to accumulate lipids and generate energy [4].

Translocase proteins, such as CD36 and fatty-acid transport proteins (FATPs), are one way that lipids can enter cells. Lipids can also enter cells via passive diffusion and FA translocation. Additionally, FA-binding proteins (FABPs) contribute to the uptake of FA [5]. De novo synthesis is the most important pathway of lipid synthesis. Citrate is the starting material for cellular lipogenesis, and the conversion of citrate to acetyl-CoA is catalyzed by ATP-citrate lyase (ACLY). FAs are produced via the synthesis of cytoplasmic acetyl-CoA, which may be derived from glucose, glutamine or acetate [6]. Acetyl-CoA forms malonyl-CoA in response to the action of acetyl-CoA carboxylase (ACACA/B). One molecule of acetyl-CoA and seven molecules of malonyl-CoA are converted to one molecule of palmitic acid (C16:0) in the presence of FA synthase (FASN). Palmitic acid can be desaturated to palmitoleic acid (16:1) via stearoyl-CoA desaturase (SCD). Palmitate can be further modified via ELOVL enzymes and FA desaturase 2 (FADS2) to generate monounsaturated fatty acids (MUFAs). Palmitic acid can also be modified by FADS2 to generate sapienate. The length of the carbon chain is subsequently increased by ELOVL enzymes. However, not all lipids can be synthesized de novo. For humans and other mammals, α-linolenic acid (ALA) and linoleic acid (LA) are essential FAs. After absorption, the FADS and ELOVL enzymes transform LA and ALA into various polyunsaturated fatty acids (PUFAs) [5].

FAO is a multistep catabolic process. Mitochondria convert long-chain FAs (LCFAs) into acetyl-CoA. To create ATP, acetyl-CoA is completely oxidized via the electron transport chain (ETC) and the tricarboxylic acid cycle (TCA). Before shuttling to mitochondria for oxidation, FAs are activated by fatty acyl-CoA synthase (ACSL) to form fatty acyl-CoA. Fatty acyl CoA is converted to fatty acylcarnitine by carnitine palmitoyl transferase 1 (CPT1) in the outer mitochondrial membrane. The inner mitochondrial membrane is dedicated to carnitine/acylcarnitine translocase, which transports acylcarnitine into the matrix of mitochondria. CPT2 reconverts acylcarnitine to acyl-CoA. To create ATP, acetyl-CoA undergoes oxidative phosphorylation (OXPHOS) in the TCA cycle [7].

Acyl-coenzyme cholesterol acyltransferase (ACAT) initiates the condensation of two acetyl-CoA molecules to generate acetoacetyl-CoA, which is the first step in the production of cholesterol. Acetyl-CoA is condensed with acetoacetyl-CoA by HMG-CoA synthetase to synthesize 3-hydroxy-3-methylglutaryl (HMG)-CoA. Mevalonate is produced by the rate-limiting enzyme involved in the production of cholesterol, HMG-CoA reductase (HMGCR). It sequentially condenses substrates to form isoprenoid farnesyl-pyrophosphate, which may be further converted to squalene via the cholesterol biosynthesis pathway and subsequently to cholesterol [8].

**Fig. 1:**
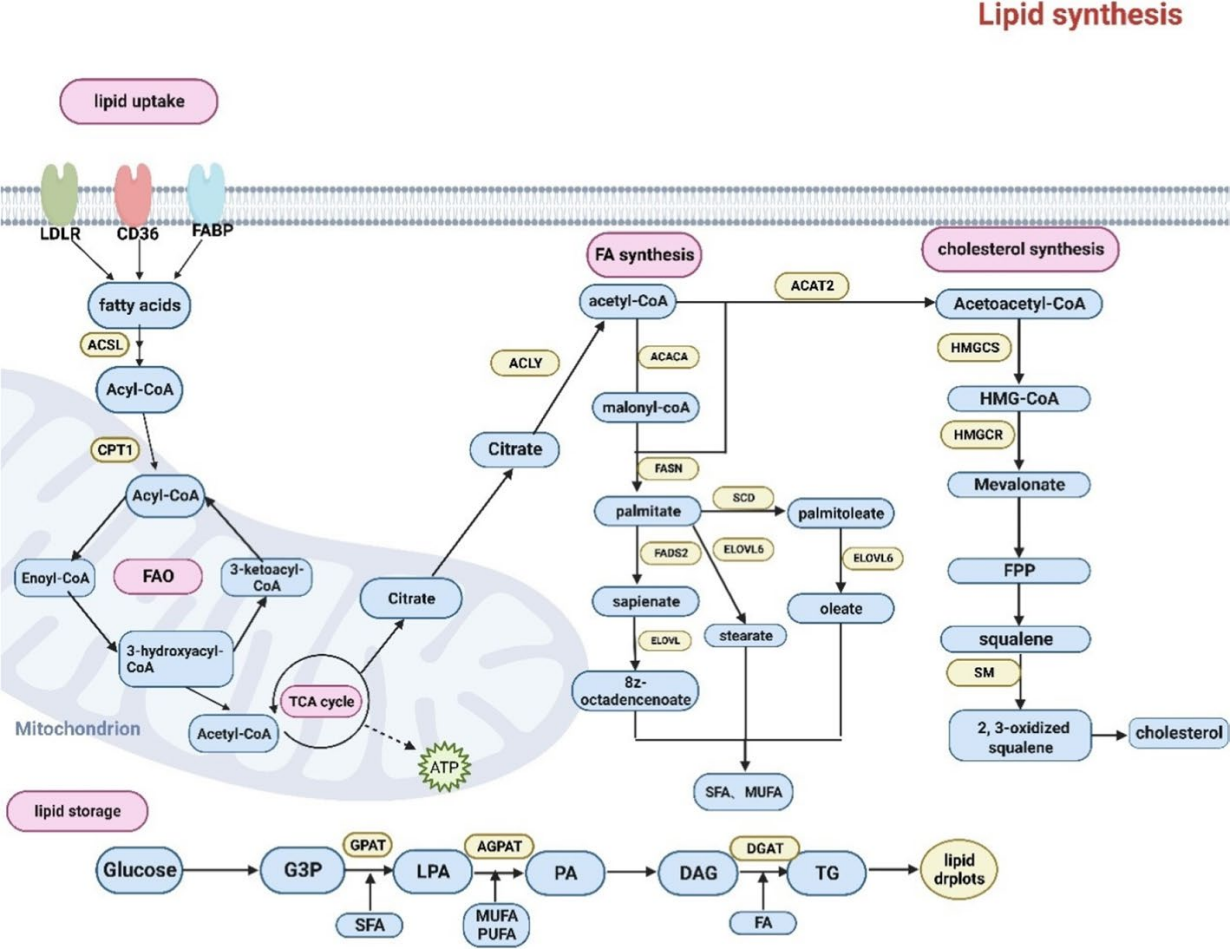
The process of lipid metabolism: Lipid metabolic processes include de novo lipogenesis, fatty acid (FA) uptake, FA oxidation (FAO), cholesterol synthesis and lipid storage

Glycerol is a common starting material for the production of diacylglycerols (DAGs) and TGs. Glycerol-3-phosphate acyltransferase, acylglycerol phosphate acyltransferase, and lysophosphatidic acid (LPA) acyltransferase work together to create DAGs. LPA and PA are the reaction intermediates. To create TGs, diacylglycerol acyltransferase enriches DAGs with more FA-CoA. Lipids are mostly stored as TGs, which are separated into lipid droplets (LDs). PL-coated vesicles store and release lipids [9] (Fig. 1).

## Reprogramming of lipid metabolism in the tumor microenvironment

### Macrophages

The three types of macrophages are activated M1 macrophages, alternatively activated M2 macrophages, and nonpolarized M0 macrophages. Although macrophages exhibit anticancer properties in the TME, TAMs are a significant subset of immune cells that promote tumor growth [10–13].

Macrophages undergo polarization and adopt the M1 phenotype, which is aerobic glycolysis dependent. M1 macrophages promote the inflammatory response and are involved in killing pathogens. In contrast, M2-TAMs are crucial for tissue regeneration, primarily via FAO and oxidative phosphorylation. Lipid metabolism regulates macrophage activation, alters macrophage phagocytosis, and promotes inflammatory responses and tumor progression [14–16].

Lipids are linked to tumor prognosis and are crucial for macrophage polarization. Long-chain FAs help myeloid cells differentiate into M2-TAMs in the lipid-rich TME, as shown by increases in CD206, IL-6, and MMP9 [17–19]. The loss of FABP5 causes macrophages to accumulate more free long-chain unsaturated fatty acids, which further enhances FAO and oxidative phosphorylation. It also activates PPAR-γ signaling, which together promote macrophage M2 polarization and aggravate allergic airway inflammation [20]. Cancer-associated fibroblasts secrete colony-stimulating factor 1 (CSF-1) to increase the ROS content in monocytes, which induces an M2-type differentiation of macrophages. The upregulation of FASN in specific mouse TAM subsets due to the secretion of CSF1 by cancer cells supports in vivo lung tumorigenesis. In this case, TAMs promote disease progression via the downstream release of the immunosuppressive cytokine IL-10 activated by PPAR-δ [21]. The levels of CD36 increase lipid uptake by TAMs. In patients with liver metastases, CD36 preferentially assigns lipid-containing vesicles to macrophages. LCFAs can be transferred from LDs to macrophage mitochondria under fluorescent conditions to provide fuel for macrophages. The CD36 gene is upregulated in metastasis-associated macrophages, and it promotes the uptake of LDs and LCFAs. CDC36 enhances the polarization of M2-TAMs via IL-4 to promote liver metastasis. Therefore, CD36 may be used as a marker for identifying liver metastasis progression in the future [22]. The receptor-interacting protein kinase 3 (RIPK3) gene is downregulated in HCC-associated macrophages, which inhibits the production of ROS and the cleavage of PPAR by caspase 1. RIPK3 stimulates FAO and PPAR activation, which causes M2-TAM production and immunosuppressive conditions. RIPK3 gene upregulation or the targeting of FAO modifies TAM functions and inhibits HCC progression [23].

Tumor cells promoted the efflux and depletion of membrane cholesterol from TAMs in a mouse model of metastatic ovarian cancer, which led to the disappearance of lipid rafts. Cholesterol efflux promotes IL-4-mediated macrophage activation and causes the disappearance of interferon(IFN)-γ signaling, which inhibits its anti-tumor effect. The deletion of ABC transporter proteins makes TAMs hyporesponsive to stimulation and promotes tumor progression. These studies suggest that membrane cholesterol efflux promotes the tumor-promoting effects of TAMs [24]. In glioblastoma, the cholesterol transporter apolipoprotein E (ApoE) accumulates around the tumor, leading to cholesterol accumulation in TAMs. It leads to Siglec-10 or PD-1 enhancement, resulting in loss of phagocytic capacity of TAMs. In addition, cholesterol accumulation impaired mitochondrial function in TAMs, and cholesterol-induced mitochondrial damage through the oxidation product 7-ketocholesterol could inhibit the phagocytosis of TAMs.ApoA1-mediated cholesterol efflux from TAMs can reduce mitochondrial damage and restore the phagocytosis of TAMs [25]. In summary, tumor cells promote cholesterol accumulation in the microenvironment. Excessive cholesterol accumulation and cholesterol efflux impair the anti-tumor effect of TAMs. Therefore, targeting cholesterol accumulation and efflux may lead to new targets for anti-tumor therapy.

### T cells

OXPHOS and FAO are the primary energy sources for T lymphocytes during their naïve and resting stages, respectively. Naïve T cells undergo antigen-stimulated differentiation into effector and memory T cells. Helper T cells (Th1, Th2, and Th17), cytotoxic T cells (CTLs), and regulatory T cells (Tregs) are examples of effector T cells (TEFs). Th cells are associated with lipid synthesis and uptake and meet their metabolic needs via glycolysis, and Tregs are associated with FAO and OXPHOS. Lipids are strongly linked to tumor stemness, metastasis, and angiogenesis and are crucial for the transformation of T-cell subpopulations. Therefore, targeting lipid metabolism in T cells enhances sensitivity to chemotherapy and immunotherapy [26].

Mature and inactivated CD8 + T cells have moderate metabolic needs, and these cells primarily produce ATP via OXPHOS [27, 28]. To maintain rapid proliferation and effector activity, activated cytotoxic T cells enhance their metabolism of glucose, glutamine, and fat. In contrast, memory CD8 + T cells increase glycolysis via OXPHOS to support adipogenesis and FAO [29]. When CD8 + TEFs identify tumor-specific antigens, they use effector molecules, such as granzymes and perforin, to destroy cancer cells. Therefore, the presence of CD8 + T cells in tumors is associated with a better prognosis in cancer patients [27, 28, 30].

Lipids have a bidirectional impact on CD8 + T lymphocytes. CD36 levels increase in tumor-infiltrating CD8 + T cells, which induces the uptake of FAs, particularly arachidonic acid (AA), to increase FAO and ferroptosis. The production of toxic cytokines (PRF1, GZMB, TBX21, and IFN-γ) is inhibited by CD36, which decreases the capacity of CD8 + T cells to fight tumors. Better anti-tumor effects are obtained when CD8 + T cells with the CD36 gene deletion are combined with an anti-PD-1 antibody [31]. CD36 gene expression in CD8 + TILs gradually increases with tumor growth. OxLDL is taken up by T lymphocytes via CD36, which promotes FAO and P38 activation and inhibits tumor necrosis factor(TNF) and IFN-γ secretion and eventually leads to CD8 + TIL dysfunction. Glutathione peroxidase 4 (GPX4) restores lipid peroxidation, rescues TNF and IFN-γ secretion, and enhances the antitumor effects of CD8 + TILs [32]. CD8 + T cells in the pancreas decrease the expression of very long-chain acyl-CoA dehydrogenase, which subsequently exacerbates very long-chain FA (VLCFA) accumulation. VLCFAs mediate lipotoxicity and impairs the mitochondrial function of CD8 + T cells in the pancreas. The frequency of abnormal mitochondria with regular cristae, a dilated intercristae space (swelling) and ultra-concentrated mitochondria with a distinct electron-dense matrix was greater in CD8 + T cells infiltrated with advanced pancreatic intraepithelial neoplasia. These changes triggered major transcriptional reprogramming in lipid metabolism and reduced FA catabolism [29, 33]. Increased lipid levels, decreased mitochondrial functions, and FAO toxicity to pancreatic CD8 + T cells led to increased cell death [34]. In summary, the excessive accumulation of lipids in CD8 + T cells impairs their antitumor properties.

Cholesterol is closely related to T-cell functions. Tumor tissues in the B16 and MC38 tumor models were rich in cholesterol. CD8 + T cells take up cholesterol after homing to tumor tissues. Cholesterol lowers GZMB, IFN-γ, and TNF-α levels and inhibits CD8 + T-cell proliferation in a dose-dependent manner by elevating ER stress in cells. Cholesterol causes immunosuppressive conditions and T-cell depletion, and it promotes an increase in PD-1, which suggests that cholesterol metabolism is a target for immunotherapy [35]. An increase in the CHOL concentration in the CD8 + T-cell plasma membrane disrupts CHOL esterification, and it may promote CD8 + T-cell proliferation. Interference with CHOL esterification using the steroid O-acyltransferase 1 inhibitor avasimibe increased the CHOL proportion in the plasma membrane of CD8 + tumor-infiltrating lymphocytes and improved effector T-cell function and proliferation [36, 37].

However, LA plays an opposite role. By promoting the development of ER–mitochondrial connections and limiting T-cell fatigue, LA improves the activity and anti-tumor potential of CTLs by converting CD8 + T cells into memory T cells [38]. Two SCFAs, butyric acid and valeric acid, increase mTOR activity in CD8 + T cells and inhibit histone deacetylase class I enzymes. SCFAs enhance the anti-tumor characteristics of CTLs by promoting the production of effector molecules, such as IFN-γ and TNF-α. A pancreatic cancer model was constructed using Panc02 pancreatic tumor cells with the type I receptor tyrosine kinase-like orphan receptor (ROR1) (Panc02/ROR1), which showed that valeric acid therapy increased the anti-tumor effectiveness of CAR-T cells in a pancreatic cancer model [39].

CD4 + T cells are multifaceted and categorized as immunosuppressive Th2 cells, ambiguous Th17 cells, pro-inflammatory and antitumor type 1 T helper (Th1) cells, or immunoregulatory T cells (Tregs) [40]. Tregs are CD4 + T cells that express FoxP3, which promotes Treg development, FA absorption, OXPHOS, and FAO [41]. Tregs prefer lipid oxidation, and lipids promote Treg production. Tregs are divided into three types: Foxp3lowCD25lowCD45RA + cells, called naïve or resting Tregs; Foxp3highCD25highCD45RA − cells, called effector Tregs (eTregs); and FOXP3lowCD25lowCD45RA − cells, called non-Tregs, which do not exhibit suppressive functions but secrete pro-inflammatory cytokines. eTregs express various markers, including CTL-associated antigen 4 (CTLA-4), PD-1, T-cell immunoglobulin and the ITIM domain, on their surfaces and exhibit increased inhibitory and proliferative abilities [41].

Inhibiting the mitochondrial lipid transporter CPT1A inhibits FAO and selectively blocks Treg differentiation. The de novo FA enzyme ACC1 controls Th17-cell development and TEF quantity and activity [42]. CD36 mediate increased Treg lipid metabolism in tumor cells, which increases Treg mitochondrial adaptability to changes in the TME and systemic metabolic demands. Although spleen Tregs were unaffected, genetic ablation of CD36 in Tregs drastically reduced lipid uptake and content in intratumor Tregs, which suggests that Tregs support increased lipid uptake by expressing CD36 [40]. I Increased lipid absorption mediated by CD36 activates the PPAR-β pathway, which improves mitochondrial fitness and the NAD/NADH ratio in Tregs [43]. Stimulation of the PPAR-β pathway may further accelerate the metabolic adaptation of intra-tumoral Tregs by increasing CD36 levels. These results suggest that CD36–PPAR-β signaling coordinates metabolic programs to support Treg persistence in the TME [44]. FABP5 deficiency reduces IL-17 production and shifts the T-cell phenotype toward the Treg phenotype FABP5 inhibition in Tregs promotes cGAS-STING-dependent type I IFN signaling, induces IL-10 production, and promotes Treg inhibitory activity. FABP5 inhibition causes changes in Treg mitochondria, decreases OXPHOS, and impairs lipid metabolism, which suggests that lipid metabolism is the target of enhanced Treg anti-tumorigenicity [45]. SREBP activity is upregulated in tumor Tregs and inhibits FASN-dependent FA synthesis. FASN is closely associated with the functional maturation of Tregs, which ultimately leads to impaired Treg activation. SREBP mediates a decrease in PD-1 in Tregs, which reduces cholesterol metabolism. Simvastatin, which targets HMGCR, inhibits PD-1 gene expression in Tregs. In summary, SREBP and FASN ultimately lead to the impaired maturation of Tregs, and SREBP inhibition promotes anti-tumor responses in Tregs [46].

Tregs interact with other immune cells to perform their tasks. Tregs prevent CD8 + T cells from secreting IFN-γ, protect mitochondrial integrity, and reduce oxidative stress, which help drive macrophage polarization toward the M2 phenotype. Treg inhibition enhances the release of CD8 + T cells, which activates AMPK signaling and SREBP inhibition. SREBP inhibition improves CD8 + T-cell capabilities, which improves anti-PD-1 anti-tumor functions [47].

### Dendritic cells (DCs)

DCs are crucial for acquired immune responses because of their ability to enhance antigen presentation to T cells. Although immature DCs depend on mitochondrial biogenesis, Toll-like receptor (TLR) activation increases glycolysis and FA synthesis, and prolonged TLR survival is often associated with decreased OXPHOS and increased glycolysis [48, 49]. TLR4 agonists include saturated FAs and polyunsaturated fatty acids (PUFAs). High- and low-density lipoproteins disrupt TLR4 signaling in mature DCs. Docosahexaenoic acid suppresses DC maturation, and lauric acid promotes T-cell activation and lipopolysaccharide-induced DC maturation [50].

DCs in the TME exhibit reduced immunological activation. The accumulation of lipids in DCs is the main cause of DC dysfunction. Reduced T-cell responsiveness and defective DCs due to diminished antigen presentation may be caused by increased LDL in tumor-associated DCs. Macrophage scavenger receptor 1 (MSR1) and CD204 are two macrophage clearance receptors [51]. DC lipid levels were reduced to normal levels, and DC functional activities were restored when DCs were treated with the ACC inhibitor 5-tetradecyloxy-2-furoic acid [52]. FA-containing tumor-derived exosomes (TDEs) promote immune dysfunction in DCs, which led to immune escape. TDE-derived FAs (not TDE-induced FA de novo synthesis) activated PPAR-α, which led to LD accumulation in DCs. It changes the metabolic preference of DCs from glycolysis to OXPHOS, which ultimately lead to immune dysfunction in DCs. Targeting PPAR-α enhanced the antitumor efficacy of PD-1 antibodies in a B16/F10 melanoma model [53]. CD36 gene expression was upregulated in Atg5-deficient DCs, which increased lipid accumulation and enhanced phagocytosis by DCs. Targeting CD36 inhibited tumor growth. Blockade of CD36 increased CD4 + T-cell initiation in Atg5-deficient DCs but did not affect CD8 + T-cell initiation or major histocompatibility complex-I presentation in DCs [54, 55].

FASN gene upregulation resulted in increased FA synthesis in ovarian cancer. High FA concentrations in the TME lead to FA accumulation in DCs, which affects their functions. The inability of DCs to elicit anti-tumor T-cell responses is caused by LD buildup. Ovarian cancer cells are generally rich in LPA, which induces PGE2 activation. DCs may be inhibited by the production of type I IFN via the PTGER4/EP4 pathway [56, 57]. DCs produce scavenging receptors in HCC and encourage the buildup of intracellular lipids, which is likely to lower T-cell activation and cytokine production [58]. DCs upregulate the expression of the CPT1A FA transporter protein in melanoma via the Wnt5a-β-catenin-PPAR-γ signaling pathway. The pathway also enhances FAO and promotes Treg development. Blockade of this pathway enhances the efficacy of anti-PD-1 antibody immunotherapy and inhibits disease progression [51]. The process of aerobic glycolysis in malignant mesothelioma cells results in high levels of lactic acid production, and high concentrations of LDs accumulate in DCs, which reduces the ability of DCs to stimulate T cells [59].

### Natural-killer (NK) cells

NK cells are the first quick responders to fundamental immune responses. Pro-inflammatory cytokines in the TME attract NK cells, which can be stimulated to attract other immune cells [60]. Their activation is consistent with increased ACLY levels and the transport of citric acid into the cytoplasm. These phenomena may be associated with epigenetic regulation and acetylation. Previous studies demonstrated that exogenous lipids impaired this metabolic process, weakened its effector function, and reduced its receptivity to stimuli, particularly in obesity [61]. The levels of additional lipid transporters and enzymes increase when NK cells take up FAs and store them in LDs to prevent lipid toxicity. This factor may limit the production of granase B and interferon production via mTORC1-mediated glycolysis, which result in diminished NK effects and insufficient function [62]. The impaired cytotoxicity of postoperative NK cells may cause recurrence and metastasis in melanoma, breast cancer, and colorectal cancer patients. Patients with colorectal cancer have two subpopulations of NK cells after surgical treatment, one of which contains high levels of the lipid transporters MSR1, CD68, and CD36, which lead to lipid accumulation [63, 64]. These NK cells cannot attack cancer cells.

Lipid-rich lung-resident mesenchymal cells transfer lipids into NK-cell exosome-like vesicles, which lead to NK-cell dysfunction and promotion of breast cancer progression and lung metastasis [65]. Cholesterol is closely related to NK cell functions, and excess cholesterol in SREBP2-driven hepatocytes induces FAO in NK T cells, which lead to NK T-cell dysfunction and suppression of their immune surveillance functions. Resulvastatin is a cholesterol inhibitor that restores NK T-cell activities [66]. CD1d-restricted invariant NK T (iNK-T) cells exert anti-tumor effects. Long-chain acylcarnitine, particularly stearoyl and palmitoyl forms, suppress immune surveillance processes, telomere damage, cell cycle arrest, and senescence in iNK-T cells in HBV-associated HCC [67].

In summary, excessive lipid accumulation in the TME exacerbates dysfunction in NK cells, which weakens their immune surveillance functions and promotes tumor progression.

### Neutrophils and myeloid-derived suppressor cells (MDSCs)

Neutrophils are recruited to the TME, where they act as macrophages and exert immunosuppressive or antitumor effects. Neutrophils promote ROS generation and increase T-cell inhibition via FAO when the glucose supply is inadequate, similar to what occurs in the TME [68]. Neutrophil-mediated tumor development, metastasis, and immunosuppression are frequently encouraged by neutrophils, despite their lethal nature. Pathologically activated neutrophils (PMNs) are called PMN–MDSCs. MDSCs undergo a metabolic reprogramming that shift their metabolic preference from glycolysis to FAO during tumorigenesis and exhibit OXPHOS during lipid accumulation in tumors. With the increased uptake of exogenous FAs by MDSCs in tumors, their immunosuppressive activities against T cells also increase [69]. Several studies showed that PMN-MDSCs in the TME died spontaneously because of ferroptosis and lipid peroxidation induced by ferroptosis-mediated suppression of T-cell functions [70].

**Fig. 2:**
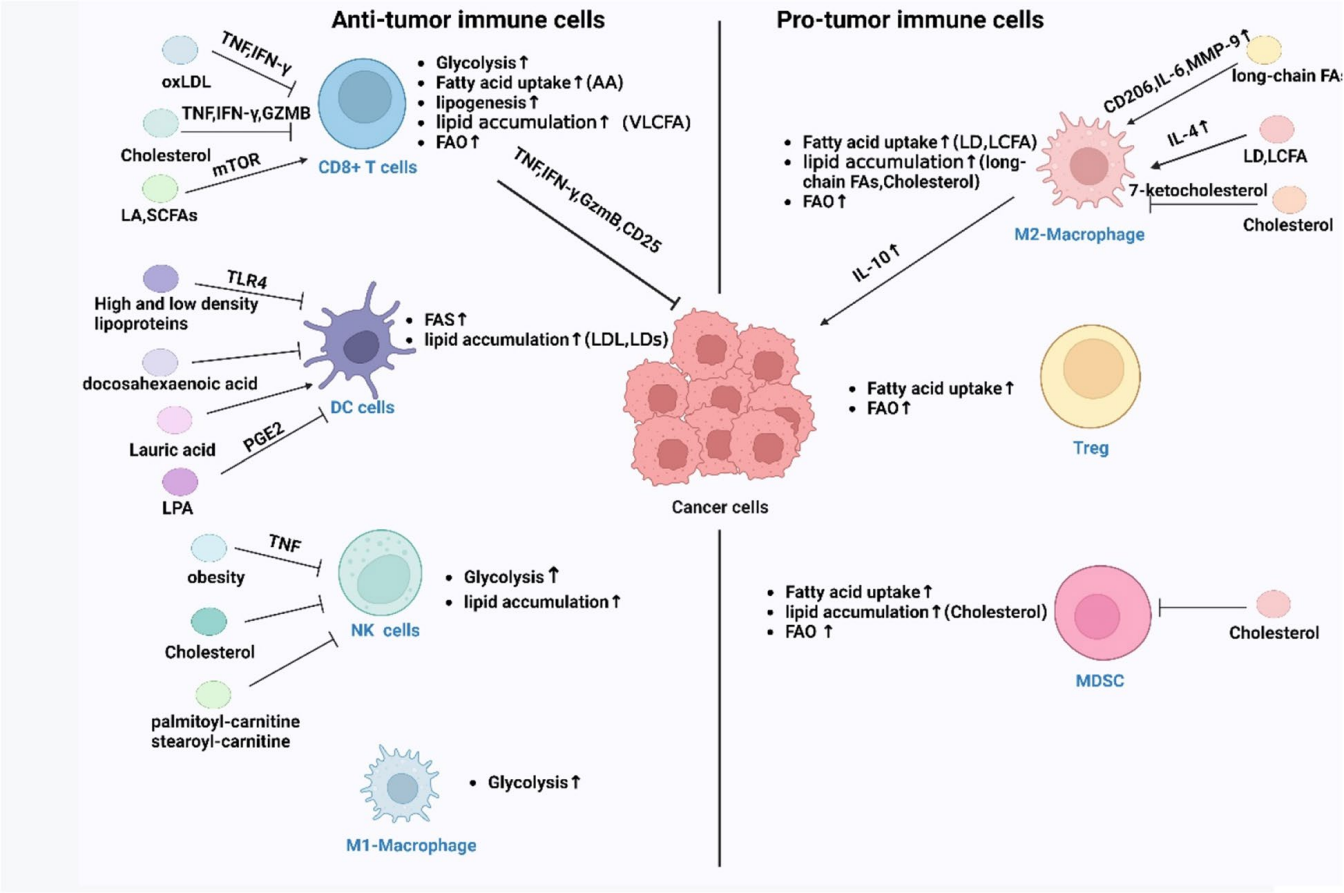
Lipid reprogram in the TME: Immune cells in tumor microenvironment showed different degrees of enhanced lipid metabolism

ApoE secreted by prostate tumor cells binds to the triggering receptor expressed on myeloid cells 2 and promotes neutrophil senescence. It enhances immunosuppressive functions and promotes tumor progression [71]. Although polymorphonuclear myeloid-derived suppressor cells (PMN-MDSCs) perform immunosuppressive tasks comparable to monocyte MDSCs, they primarily block immune responses to particular antigens by nitrifying ROS on T-cell receptors [72]. Increased FATP2 expression and fat accumulation have been observed in PMN-MDSCs derived from colon, pancreas, and lymphatic system cancers. FATP2 knockdown inactivated PMN-MDSCs by suppressing CD8 + T cells, which indicated that PMN-MDSCs used FAs from the TME [73, 74]. The number of total TGs, especially TGs carrying AA, in PMN–MDSCs isolated from the spleens of FATP2-knockout mice was reduced. This phenomenon was recently observed in PMN-MDSCs via other FA transporters, such as CD36. FAO may immunosuppress PMN-MDSCs via ROS-producing T cells [73, 74]. X-box binding protein 1 (XBP1) promotes cholesterol synthesis and secretion, and this cholesterol is internalized by MDSCs in the form of small-cell vesicles to cause immunosuppression. Lowering cholesterol levels or targeting XBP1 enhances the antitumor responses of MDSCs [75] Fig. 2.

### Cancer-associated fibroblasts (CAFs)

As a heterogeneous and plastic population, CAFs are crucial for tumor development, survival, and metastasis. CAFs produce several growth factors, chemokines, and cytokines that promote tumor growth, angiogenesis, and immunosuppression and are involved in the metabolism of tumor cells. CAFs also modify the TME to provide the nutrients required for tumor metabolism via lactic acid, amino acid, and lipid metabolism [76, 77]. A noteworthy metabolic change occurs in CAFs when they move from lipid synthesis to lipid secretion [78]. By delivering lipids to surrounding tumor cells, CAFs feed cancer cells, which contributes to their growth and metastasis. Astrocytic conversion to CAFs in pancreatic cancer cells triggers the release of lysophosphatidylcholine (LPC), alterations in intracellular lipids, and the subsequent stimulation of phosphatidylcholine production and cell membrane construction [79]. Autologous chemokines and extracellular enzymes hydrolyze LPC to create lysophosphatidic acid, which is a potent pro-mitotic and wound-healing agent that accelerates tumor growth. FATP1 expression is upregulated in cancer cells after exposure to CAF-conditioned media, which lead to increased FA absorption. CAFs induced FATP1 upregulation in human triple-negative breast cancer cells, which increased the intake of exogenous FAs in the TME [60]. CAFs also transfer lipids to cancer cells via the extracellular space, which promotes cancer cell proliferation [77]. Ox-LDL promoted lipid peroxidation of CD36 + CAFs, p38 phosphorylation, and CEBPA/D binding to the migration inhibitory factor (MIF) promoter in HCC. It ultimately promoted macrophage MIF secretion in a CD36-dependent manner. MIF enhances the immunosuppressive effect and tumor stemness of MDSCs, which together promote the progression of HCC. Palmitic acid-induced lipid accumulation in HCC cells promotes the immunosuppressive phenotype of co-cultured macrophages and fibroblasts [80]. PDAC cells directly targeted ACSL4 after the uptake of miR-3173-5p and reduced lipid ROS levels. Although miR-3173-5p controlled GEM chemoresistance in PDAC tumors, it did not stimulate tumor development in vivo [81]. Hypoxia-inducible factor-1α (HIF-1α) induces SCD1 to increase the number of lipid droplets inside CAFs. The inhibition of SCD1 reduces the number of lipid droplets and inhibits lung tumor growth. The HIF-1α/SCD1 axis controls the buildup of LDs in CAFs [82] (Table 1).

**Table 1:** Reprogramming of lipid metabolism in the TME and it plays an important role in diseases

Immune cells	Indicators of lipid metabolism	Mechanisms	Diseases	References
TAM	FABP5	Enhance FAO, oxidative phosphorylation and activate PPAR-γ signaling	Allergic airway inflammation	[20]
	FASN	Promote PPAR-δ activation and IL-10 release	Lung cancer	[21]
	CD36	Promote the uptake of LDs and LCFAs. Enhance the polarization of M2-TAMs	Liver metastases	[22]
	PPAR	Promote FAO and the synthesis of M2-TAMs	HCC	[23]
	25-hydroxycholesterol	Activate TLR4 and nuclear factor-κB signaling	Atherosclerosis	[83]
	APOA1 ABCG1	Promote cholesterol efflux	Atherosclerosis	[84]
	Asprosin	Activate the p38/Elk-1 axis. Promote cholesterol efflux and inhibit lipid accumulation	Atherosclerosis	[84]
CD8 + T	CD36	Promote uptake of FAs and FAO. Inhibit the release of IFN-γ, PRF1, GZMB, and TBX21 Promote uptake of oxLDL and P38 activation	-	[31, 32]
	very long-chain acyl-CoA dehydrogenase	Increases the very long-chain FA accumulation and FAO	PDAC	[39]
	Cholesterol	Increasing ER stress and decrease GZMB, IFN-γ, and TNF-α levels	B16 and MC38 tumor models	[35]
	LA	Enhance the formation of ER–mitochondrial contacts	-	[38]
	SCFAs	Promote the production of CD25, IFN-γ and TNF-α	Pancreatic cancer model	[39]
Treg	CPT1A	Promote FAO and Treg differentiation	-	[42]
	CD36	Activate the PPAR-β pathway and promote lipid uptake	-	[40]
	FABP5	increase OXPHOS and promote lipid metabolism	-	[45]
	SREBP、FASN	inhibit FA synthesis and reduce cholesterol metabolism	-	[46]
DC cells	ACC	Increase the amount of DC lipids	-	[52]
	TDE、PPAR-α	Promote OXPHOS	B16/F10 melanoma model	[53]
	CD36	Increase lipid accumulation and phagocytosis by DCs	-	[54, 55]
	FASN、LPA	Increased FA synthesis and lead to FA accumulation in DCs	Ovarian cancer	[56, 57]
	Scavenging receptors	Promote lipid accumulation and decrease cytokine levels	HCC	[58]
	CPT1A	Enhance FAO and promotes Treg development	Melanoma	[51]
	LDs	Decrease the ability of DCs to activate T cells	Malignant mesothelioma	[59]
NK cells	lipid transporters and enzymes	Inhibit granase B and interferon production. Diminish NK effects	-	[62]
	MSR1 CD6 CD36	lipid accumulation	Melanoma, breast, colorectal cancer	[63, 64]
	lipids	Transfer lipids into NK-cell exosome-like vesicles and lead to NK cell dysfunction	Breast cancer and lung metastasis	[65]
	Cholesterol SREBP2	Induce FAO	-	[66]
	long-chain acylcarnitine	Promote iNK T-cell senescence, cell cycle arrest, and telomere damage	HBV-associated HCC	[67]
MDSCs	ApoE	Promotes neutrophil senescence	Prostate tumor	[71]
PMN-MDSCs	FATP2	Increase lipid accumulation	Colon, pancreas, and lymphatic system cancers	[73, 74]
	XBP1	Promotes cholesterol synthesis and secretion	-	[75]
CAFs	LPC	Create lysophosphatidic acid and accelerate tumor growth	PDAC	[79]
	FATP1	Increase the intake of exogenous FAs	Triple-negative breast cancer	[60]
	CD36	promotes lipid peroxidation and p38 phosphorylation,	HCC	[80]

## Lipid metabolism and cancer therapy

### Lipid metabolism and chemotherapy, radiotherapy

Chemoresistance in cancer cells is characterized by reduced fluidity of lipid bilayers in the membrane, which results in impaired uptake of drugs via passive diffusion or endocytosis. For example, CPT1B mRNA levels are elevated in recurrent breast cancer tissues, and CPT1B expression is elevated in chemotherapy-resistant breast cancer patients. The number of lipid droplets is increased in chemotherapy-resistant breast cancer cells [85]. Methyl-β-cyclodextrin (MCD), a cholesterol-depleting agent, has a sensitizing effect on a variety of chemotherapeutic drugs. MCD activates the FasR/FasL pathway via p53 and increases the entry of doxorubicin into the nucleus to promote cell death. MCD combined with adriamycin slowed the growth of tumors in mice. MCD enhanced tamoxifen-induced anticancer effects by causing cell cycle arrest and inducing apoptosis. Exogenous cholesterol supplementation abrogated the combined anticancer effects of tamoxifen and MCD [86, 87]. Resistin is secreted primarily by adipocytes. It induces colorectal cancer cells to arrest in the G1 phase, and cells exposed to resistin become resistant to 5-fluorouracil chemotherapy [88].

Exogenous supplementation of monounsaturated and bisounsaturated fatty acids improved the radiosensitivity of cervical cancer cells. This effect upregulated PPAR-γ and P53 signaling, which promoted increased fatty acid uptake by tumor cells [89]. Targeting CPT1A activated mitochondrial apoptosis in vitro and in vivo in NPC patients. It promoted sensitivity to radiotherapy in NPC patients [3]. Arachidonic acid produced enzymes, such as cytosolic phospholipase A2, cyclooxygenase and lipoxygenase, which caused tumor resistance to radiotherapy and led to the failure of tumor treatment [90].

In summary, the role of lipids in tumor radiotherapy and chemotherapy is complex. In general, tumor lipid reprogramming often leads to tolerance to chemotherapy and radiotherapy so the tumor can escape the disappearance of lipids. There are also many lipid inhibitors in clinical practice, and their combination with chemotherapy and radiotherapy often results in improved therapeutic effects.

### Lipid metabolism and immunotherapy

Tumor-infiltrating CD8 + T lymphocytes absorb FA via CD36, which also causes ferroptosis and lipid peroxidation in the TME. Immunotherapy with anti-PD-1 antibodies is more effective against tumors when combined with CD36 blockade. CD36 enhances mitochondrial adaptation by activating the PPAR-β pathway, which allows Tregs to adapt to a lactic acid-rich TME [91]. The quantity of CD36 + CAFs was inversely associated with PD-1 sensitivity in a mouse model of HCC. When coupled with anti-PD-1 therapy, CD36 inhibitors had greater anti-tumor effects than when used alone. Combination therapies also change the type of immune cells. The proportions of Tregs and MDSCs decreased, and the proportion of CD8 + T cells increased [82]. Research indicated that FABP5 diminished the pace of β-oxidation, which resulted in the buildup of LDs in monocytes and encouraged the production of the IL-10 gene. IL-10 upregulates PD-1 gene expression on Tregs via the JNK–STAT1 pathway [92]. Tissue-resident memory T (Trm) cells indicate a better prognosis in gastric cancer patients. Targeting PD-L1 lowered the expression of FABP4 and FABP5 in gastric cancer cells, and Trm cells express greater amounts of PD-1. These changes promotes lipid uptake by Trm cells, which increase the number of Trm cells. This finding suggested that lipid metabolism promotes Trm cell survival and contributes to the sensitivity of gastric adenocarcinoma to immunotherapy [93]. MSI and tumor mutational load strongly correlated with FASN in a previous study. The expression of FASN exhibited a negative correlation with the levels of PD-1 and PD-L1, which suggest that FASN is a target for anti-PD-1 therapy. FASN was associated with immune cell infiltration in bladder cancer, and FASN inhibition promoted the sensitivity of tumors to ICI therapy [94]. SCD1 inhibition promoted CCL4 gene expression by downregulating Wnt/β-linked protein signaling, enhancing CD8 + TEF production and lipid accumulation, and promoting DC recruitment. Anti-PD-1 antibodies and SCD1 inhibitors work together to improve anti-tumor effects [95]. ACAT1 inhibition promoted the proliferation of CD8 + T cells and increased plasma membrane cholesterol levels. Together, anti-PD-1 antibodies and ACAT1 inhibitors have shown increased anti-tumor effectiveness [96]. Anti-PD-1 immunotherapy is not highly effective at treating nasopharyngeal cancer, despite the reality that CD8 + T cells are prevalent in the immune environment. Recent studies showed that nasopharyngeal carcinoma cells promoted Treg cell development via CD20-CD70. CD70 knockout inhibited cholesterol homeostasis and fatty acid metabolism in Treg cells. CD70 blockade synergized with an anti-PD-1 antibody to further enhance the immunotherapy efficacy in nasopharyngeal carcinoma [97]. Benzofibrate is an agonist of the PGC-1α/PPAR complex, and it enhanced the tumoricidal effect of PD-1 blockers. It also increased FAO and mitochondrial respiratory capacity and upregulated CPT1 and Bcl2, which prevented CTL apoptosis and further increased the anti-tumor effects of PD-1 blockade [98]. I In tumor-infiltrating (TI) Treg cells, the PD-1 signaling pathway can promoted lipid metabolism. Inhibition of PD-1 reduced the metabolic fitness of Tregs and enhanced the anti-tumor effect [99]. MiR-21-3p promotes lipid peroxidation by enhancing ROS generation, which promotes IFN-mediated ferroptosis in melanoma. The combination of miR-21-3p overexpression and an anti-PD-1 antibody was used in combination [100]. Gut bacteria that produce SCFAs are positively associated with anti-PD-1/PD-L1 responses in gastrointestinal cancers [101]. Type I IFNs enhance the exhaustion of terminal CD8 + T cells in tumors. IFN-I disrupted CD8 + T-cell lipid metabolism, which resulted in the aberrant accumulation of lipids and oxidative stress. IFN-I stimulation is associated with a poor response to anti-PD-1 therapy [102].

In summary, the targeting of crucial enzymes involved in lipid metabolism impacts T-cell and DC activities, which further influences the sensitivity to ICB treatment. Lipid metabolism also plays an essential role in PD-1/PD-L1 sensitivity.

### Lipid metabolism and inhibitors

Many drugs target lipid reprogramming. However, only a few drugs have reached clinical trials. We summarize the common drugs that target fatty acid metabolism.

The CD36 inhibitors include JC61.3, FA6.152, and SSO. CD36 inhibitors improve the antigen presentation capacity of DCs. The inhibition of the PPAR pathway also decreases the number of Treg cells and upregulates PD-1, which improve the function of CD8 + T cells [103]. JC61.3 inhibited FA and LDL protein uptake, impeded the progression and dissemination of gastric and oral cancer [104]. The oleic acid analog sulfo-n-succinimidyl oleate (SSO) irreversibly bound to CD36. It also effectively inhibited the progression of cervical cancer [105]. FABP is also an important target for lipid uptake. The FABP inhibitors SBFI-26 and BMS3094013 inhibited prostate cancer and ovarian cancer. The sensitivity of breast cancer to doxorubicin may be increased by targeting FABP. BMS309403 improved the effectiveness of carboplatin by preventing tumor spread in an in vivo model of ovarian cancer [106].

FASN is an important target of lipid metabolism. The FASN inhibitors used included orlistat, TVB-3166, TVB3664, TVB2640 and Fasnall. FASN inhibitors can decrease ROS, IL-10, and TNF-α production while preventing Treg cell activation [1]. Orlistat effectively inhibited the progression of prostate cancer in mice. Orlistat caused ferroptosis and lipid peroxidation in lung cancer cells [107]. TVB-3166 and TVB-3664 promote tumor cell apoptosis and inhibit tumor progression via the PI3K-Akt-mTOR pathway and β-catenin signal transduction. The combination of these two inhibitors with chemotherapy achieved better anti-tumor effects. It played an anti-tumor role in colon cancer, lung cancer, liver cancer and oral squamous cell carcinoma [108]. Clinical trials of TVB-2640 in astrocytoma and nonalcoholic fatty liver disease have been completed (NCT03808558, NCT02223247, NCT02980029, NCT03179904, NCT03032484, and NCT03938246). TVB-2640 had anti-tumor effects in high-grade astrocytoma, ovarian cancer and breast cancer. Fasnall promoted apoptosis and inhibited tumor progression in the MMTV-Neu breast cancer mouse model [109].

ETC-1002 is a small molecule inhibitor of ACLY. ECT-1002 inhibits lipogenesis by activating the AMPK pathway, which plays an important role in liver cancer. ETC-1002 is currently undergoing clinical trials for the treatment of hypercholesterolemia (NCT05687071, NCT05683340, and NCT04784442). SB-204990 attenuates cisplatin resistance by inhibiting ACLY to activate the AMPK-ROS pathway. Therefore, this approach has become an effective strategy for the treatment of ovarian cancer [110].

AThe ACC inhibitors include PF-05221304, ND-646, and ND-654. PF-05221304 effectively reduced liver fat and played a role in non-alcoholic fatty liver disease. ND-646 inhibited fatty acid synthesis and tumor growth in non-small cell lung cancer. ND-646 alone or in combination with carboplatin inhibited the progression of non-small-cell lung cancer(NSCLC) in a mouse model [111]. ND-654 inhibited the progression of hepatocellular carcinoma and increased the survival rate of tumor-bearing mice when combined with sorafenib [112].

SCD1 has been clearly associated with a variety of tumors, and inhibitors against SCD1 are very popular in clinical practice. A939572 effectively induced endoplasmic reticulum stress in renal clear cell carcinoma and inhibited tumor progression [113]. A939572 combined with temsirolimus inhibited tumor growth. A939572 inhibit3e epithelial–mesenchymal transition and EGFR/PI3K/AKT signaling in lung cancer and inhibited its progression. MF-438 effectively downregulated YAP/TAZ. MF-438 combined with a MAPK inhibitor effectively reduced tumor drug resistance MK-8245 is a potent liver-targeted SCD inhibitor with antidiabetic and antidyslipidemic effects. It has been tested in type 2 diabetes clinical trials (NCT00972322) [1]. CAY10566 is a specific SCD inhibitor that reduces hepatic steatosis and hepatic lipid accumulation via activation of the AMPK pathway and lipophagocytosis [114].

DGATs play an important role in fatty acid storage. The known inhibitors of DGAT1 include A922500, AZD3988 and AZD7687. A922500 reduces postprandial triglycerides and cardiovascular risk. A922500 also inhibited triglyceride synthesis in plays a therapeutic role in prostate cancer. [115]. AZD7687 successfully reduced the number of circulating TAG in clinical trials. Clinical trials for type 2 diabetes and obesity have been completed (NCT01217905). [116]. The inhibitors of DGAT2 include PF-06424439, JNJ-DGAT2-A and JNJ-DGAT2-B. PF-06424439 reduced LD formation in gastric cancer. It also enhanced the sensitivity of breast cancer to cisplatin and doxorubicin chemotherapy. Breast cancer cells treated with PF-06424439 exhibited radiosensitivity [1] (Table 2).

**Table 2:** Drugs targeting lipid metabolism and their mechanisms

Target	Drugs	Tumors	mechanisms	Clinical trials
CD36	JC61.3	oral cancer gastric cancer	inhibit FA and LDL protein uptake; prevents lipid droplet formation	N/A [104]
	FA6.152	oral cancer	prevent lipid droplet formation	N/A [1]
	SSO	cervical cancer	inhibit FA uptake	N/A [105]
FABP	SBFI-26	prostate cancer	inhibit lipogenesis	N/A [1]
	BMS3094013	ovarian cancer	inhibit lipogenesis	N/A [106]
FASN	Orlistat	prostate cancer NSCLC	induce lipid peroxidation and ferroptosis	N/A [107]
	TVB-3166	lung cancer ovarian cancer prostate cancer HCC	promote tumor cell apoptosis	N/A [108]
	TVB-3664	colon cancer lung cancer	promote tumor cell apoptosis	N/A [108]
	TVB2640	ovarian cancer breast cancer	reduce hepatic fat	NCT03808558 NCT02223247 NCT02980029 NCT03179904 NCT03032484 NCT03938246 [109, 117]
	Fasnall	breast cancer	promote apoptosis	N/A [109]
ACLY	ECT-1002	HCC	inhibit lipogenesis	NCT05687071 NCT05683340 NCT04784442 [110]
	SB-204990	ovarian cancer	inhibiting ACLY and activate the AMPK-ROS pathway	N/A [110]
ACC	PF-05221304	-	reduce liver fat	N/A [111]
	ND-646	NSCLC	inhibite fatty acid synthesis	N/A [111]
	ND-654	HCC	inhibit lipogenesis	N/A [112]
SCD1	A939572	lung cancer ovarian cancer	induce endoplasmic reticulum stress; inhibit epithelial-mesenchymal transition; inhibit EGFR/PI3K/AKT signaling	N/A [113]
	MF-438	NSCLC ovarian cancer melanoma	downregulate YAP/TAZ	N/A [1]
	MK-8245	-	-	NCT00972322 [117]
	CAY10566	-	activate the AMPK pathway and lipophagocytosis	N/A [114]
DGAT1	A922500	cervical cancer HCC	inhibit triglyceride synthesis	N/A [115]
	AZD3988	prostate cancer	decrease TAG synthesis	N/A [1]
	AZD7687	-	reduced circulating TAG	NCT01217905 [116]
DGAT2	PF-06424439	gastric cancer breast cancer	prevents LD formation	N/A [1]
	JNJ-DGAT2-A/B	-	inhibit TG synthesis	N/A [1]

### Lipid nanoparticles are great delivery systems

Lipids are polar and fat soluble, which makes them excellent drug carriers. Lipid nanoparticles (LNPs) are generally composed of ionizable lipids, cholesterol, phospholipids and polyethylene glycol lipids. LNPs are crucial for the use of vaccines and cancer treatment. Liposomes are among the most widely used drug delivery systems. These nanoparticles are currently the only nanoparticle systems approved by the FDA for clinical use [118]. The advantage of LNPs is that they can change the pharmacokinetics to increase the drug concentration in tumor tissues and improve the anti-tumor effects [119]. IVTIL-15 mRNA may be introduced into colorectal cancer cells via a liposome/protamine system (CLPP) nano-delivery method. This system significantly reduced tumor formation and metastasis. It also enhanced lymphocyte activity and promoted the number of CD8 + T cells, which further plays a role in immunotherapy [120]. The combination of PTEN mRNA nanoparticles (mPTEN@NPs) successfully promoted the infiltration of CD8 + T and CD3 + T cells in a Pten-deficient mouse model of prostate cancer. It also promoted anti-tumor effects. Several researchers developed a therapy called CATCH that combines lipid nanoparticle-mRNA formulations with dendritic cell therapy. This therapy promotes the release of a variety of cytokines and chemokines and activates dendritic cells. It also promotes the anti-tumor capacity of T cells. The downregulation of CCL5 by the immunoregulatory factor 5 (IRF5) mRNA/C–C chemokine ligand 5 (CCL5) siRNA (LPR) nanoparticle combination (LPR@CHG) significantly increased M1-TAMs and modified the immune microenvironment in pancreatic cancer [121]. In summary, LNPs increase the drug concentration in tumor tissue and reduce adverse reactions. It also further changes the immune microenvironment and promotes the formation of an anti-tumor immune microenvironment.

## Strengths and limitations

The advantages of this review are as follows: 1. The lipid metabolism reprogramming of immune cells in the tumor microenvironment is systematically illustrated. It indicates that the changes of lipid metabolism in the immune microenvironment provide favorable conditions for the survival of tumor cells and promote the formation of immunosuppressive microenvironment during tumor development. 2. A comprehensive description of the relationship between lipid metabolic reprogramming and tumor treatment. We explored the mechanisms by which reprogramming of lipid metabolism promotes tumor resistance to radiotherapy, chemotherapy, and immunotherapy. 3. Different agents targeting tumor lipid metabolism are described. We also describe the roles of LNPs in different tumors, the specific mechanisms and clinical trials. Besides, lipid is a good carrier and the important role of LNPs is also described. In summary, we summarize the reprogramming of lipid metabolism in the immune microenvironment and its implications for tumor therapy, and describe drugs that target lipid metabolism. Of course, our study also has some limitations: 1. The lack of changes in tumor cell lipid metabolism. Although the immune microenvironment is critical for tumor progression, the direct metabolic alterations of tumor cells have a direct role in tumor initiation and progression, which we did not mention. 2. Although we have described the drugs targeting lipid metabolism, many drugs do not work in the human body. The reasons for this are complicated and lack of mature conclusions.

## Summary

Lipids are involved in diverse fundamental processes of cell biology, such as proliferation, differentiation, migration, stress response, and cell death. With the increase in the global obesity rate, research on the increase in microenvironmental fatty acids and adipokines has increased. Obesity is a metabolic disease that is characterized by "systemic lipid metabolism reprogramming". Obese cancer patients have reduced survival and are resistant to treatment. Cancer cells promote the absorption and oxidation of fatty acids by adipocytes via lipolysis and the transfer of fatty acids to their own cells. Adipokines released by adipocytes, such as IL-6 and leptin, are crucial for the promotion of chemotherapeutic resistance in cancer cells. Obesity affects the efficacy of anti-PD-1 therapy in cancer patients. Obesity causes an increase in PD-1 expression and tumor dysfunction by hastening T-cell senescence [122]. Chemotherapy is still the first-line therapy for cancer treatment. Chemoresistance is related to a variety of factors, including cell proliferation, apoptosis, DNA damage and epithelial-mesenchymal transition [123, 124]. A number of studies have shown that lipid droplets and phospholipids play an important role in tumor chemotherapy resistance in breast cancer and other tumors. The crosstalk between breast cancer and its microenvironment can further affect tumor growth and metastasis [125, 126].

Lipids significantly influence tumor metastasis and developments. Novel approaches for the treatment of tumors may be found by focusing on lipid metabolism. A variety of inhibitors and drugs have been developed to target lipid uptake, lipogenesis, fatty acid oxidation and lipid storage. These inhibitors have clear anti-tumor effects, and some of these agents have entered clinical trials [127]. Immunotherapy has changed the traditional model of cancer treatment via the application of PD-1/PD-L1 and CTLA4 in many cancer patients. However, only a portion of these patients benefited from these inhibitors. An increasing number of clinical studies demonstrated that a large proportion of first responders eventually developed recurrence after several months or years and exhibited fatal disease resistance. Tumor cells can evade T-cell immune monitoring due to the many alterations inside the tumor. Immune cells that infiltrate tumors typically experience metabolic stress as a result of the dysregulation of the metabolic activity of tumor cells, which impairs anti-tumor immune responses [128].

Although many inhibitors targeting lipid metabolism have been developed, few of these agents have been tested in preclinical trials. This lack of testing may be attributed to the low membrane penetration efficiency and difficulty in achieving an effective blood concentration near the tumor. However, targeting lipid metabolism combined with immunotherapy remain the focus of our continued exploration. Excessive lipid accumulation causes immune cell dysfunction, which is why immunotherapy rarely benefits people. A number of studies have shown that the targeting of lipid metabolism enzymes, such as FASN and CD36, restored the anti-tumor effect of CD8 + T cells and enhanced the effect of immunotherapy. These findings may lead to new ideas for tumor treatment [129, 130].

Lipids are excellent drug carriers due to their polarity and lipid solubility. Liposomes and exosomes are good drug carriers. Liposomes are among the most widely used drug delivery systems and are currently the only nanoparticle system approved by the FDA for clinical use. Liposome-encapsulated drugs promote efficient target gene silencing during glycolysis and chemotherapy. It enhances the antiproliferative ability of cells and has a better anti-tumor effect on non-small cell lung cancer [131]. In addition, extracellular vesicles secreted by TME promote communication between tumor cells and play an important role in breast cancer metastasis and chemotherapy resistance [132].

In summary, lipid metabolism is an important metabolic pathway for tumor development. It is worth exploring the crosstalk between lipids and the immune microenvironment. Targeting lipid metabolism combined with immunotherapy also provides a new direction for tumor treatment.

## Data Availability

Not applicable.

## Abbreviations

TG: Triglycerides
VLDL: Very low-density lipoprotein
LPL: Lipoprotein lipase
FA: Fatty acid
LDs: Lipid droplets
FABPs: FA-binding proteins
FASN: FA synthase
ACC: Acetyl-CoA carboxylase
SCD: Stearoyl-CoA desaturase
MUFAs: Monounsaturated fatty acids
ALA: α-Linolenic acid
LA: Linoleic acid
PUFAs: Polyunsaturated fatty acids
LCFAs: Long-chain FAs
ACSL: Fatty acyl-CoA synthase
OXPHOS: Oxidative phosphorylation
TNF: Tumor necrosis factor
MSR1: Macrophage scavenger receptor 1
ACC: Acetyl-CoA carboxylase
SM: Squalene monooxygenase
CPT1: Carnitine palmityl transferase 1
ACLY: ATP citrate lyase
FAO: Fatty acid oxidation
TAMs: Tumor-associated macrophages
PPP: Pentose phosphate pathway
ECM: Extracellular matrix
HA: Hyaluronic acid
CSF1: Colony stimulating factor 1
PPAR: Peroxisome proliferator activated receptor
FABP4: Fatty acid binding proteins 4
PGE2: Prostaglandin E2
CTLS: Cytotoxic T lymphocytes
Th1: Pro-inflammatory T cells 1
Th2: Immunosuppressive T cells 2
Th17: Ambiguous T cells 17
Tregs: Immunoregulatory T cells
CPT1A: Carnitine palmitoyltransferase-1A
TLR: Toll-like receptor
XBP1: X-box binding protein 1
CAF: Cancer-associated fibroblasts
LPC: Lysophosphatidylcholine
FATP1: Fatty acid transporter 1
ECs: Endothelial cells
VEGF: Vascular endothelial growth factor
GBM: Glioblastoma
GPX4: Glutathione peroxidase 4
IFN-γ: Interferon-γ
LTB4: Leukotriene B4
PGE2: Prostaglandin E2
25-HC: 25-Hydroxycholesterol
ABCG1: ABC protein G1
LXRs: Liver X receptors
HCC: Hepatocellular carcinoma
RIPK3: Receptor-interacting protein kinase 3
CAR-T: Chimeric antigen receptor-T
ICI: Immune checkpoint inhibitor
PD-1: Programmed cell death protein 1
LNPs: Lipid nanoparticles
NSCLC: Non-small-cell lung cancer
